# Editorial: Interplay of genetics and environment in pediatric diabetes: insights and innovations

**DOI:** 10.3389/fendo.2025.1720343

**Published:** 2025-10-17

**Authors:** Tiago Jeronimo Dos Santos, Marina Ybarra, Rade Vukovic, Agata Chobot

**Affiliations:** ^1^ Department of Nursing Sciences, Physiotherapy, and Medicine, Faculty of Health Sciences, University of Almería, Almería, Andalusia, Spain; ^2^ Vithas Almería, Instituto Hispalense de Pediatría, Almería, Spain; ^3^ Department of Pediatrics, Children’s Hospital, London Health Science Center, Western University, London, ON, Canada; ^4^ Department of Pediatric Endocrinology, Mother and Child Health Care Institute of Serbia, Belgrade, Serbia; ^5^ Faculty of Medicine, University of Belgrade, Belgrade, Opole, Serbia; ^6^ Department of Pediatrics, Institute of Medical Sciences, University of Opole, Opole, Poland; ^7^ Department of Pediatrics, University Clinical Hospital in Opole, Opole, Poland

**Keywords:** pediatric diabetes, type 1 diabetes, type 2 diabetes, obesity, genetic predisposition, environmental factors, metabolic syndrome, insulin resistance

## Introduction

The field of pediatric diabetes is rapidly evolving while being shaped by the complex interplay of genetic and environmental factors. Type 1 diabetes (T1D), long considered a classic autoimmune disease of childhood, now shows a more heterogeneous course with adult-onset forms and links to obesity. Meanwhile, type 2 diabetes (T2D), traditionally confined to adults, is rising sharply in adolescents, driven by the obesity epidemic and disproportionately affecting minority groups. These shifts challenge traditional classifications and highlight the need for a more nuanced understanding of diabetes pathogenesis and management in children and adolescents.

## Aims and objectives of the Research Topic

The aim of this Research Topic was to explore new scientific findings regarding the interplay between genetic predisposition and environmental factors in the development of pediatric diabetes and metabolic syndromes. While T1D is usually regarded as an autoimmune condition and T2D as a metabolic disorder, their boundaries in children and adolescents are increasingly blurred by overlapping influences such as obesity, insulin resistance, lifestyle, and psychosocial determinants.

Our objective was twofold: first, to synthesize emerging evidence that highlights how both genetic factors and modifiable exposures, such as diet, sleep, microbiome, and obesity contribute to the heterogeneity of pediatric diabetes; and second, to identify potential pathways for improved diagnosis, prevention, and management in young populations. By integrating perspectives from molecular genetics, clinical endocrinology, and public health, this Research Topic sought to advance precision medicine approaches and provide actionable insights for clinicians and researchers working to reduce the growing burden of diabetes in children and adolescents.

## Thematic overview of contributions

The articles published within this Research Topic collectively highlight the multidimensional nature of pediatric diabetes, where both genetic predisposition and environmental influences act as overlapping drivers of disease development and progression. Rather than examining these forces in isolation, the contributions emphasize how their interplay shapes clinical presentation, metabolic outcomes, and therapeutic challenges.

Environmental and lifestyle determinants emerged as a critical theme. A systematic review by Porri et al. explored the role of sleep duration and quality in the prevention and treatment of childhood obesity. Sleep disturbances, often overlooked in clinical practice, were consistently associated with adverse metabolic outcomes, including higher risks of obesity and insulin resistance. As obesity represents a shared pathway contributing to T2D, while less directly to T1D risk, this review highlights sleep as a modifiable behavioral factor with significant implications for pediatric endocrinology. Complementing this, a narrative review by Badr et al. examined the broad endocrine consequences of childhood obesity. The review detailed how obesity is not only a risk factor for T2D but also disrupts hormonal regulation across multiple axes (thyroid, gonadal, adrenal, and bone metabolism) further compounding the metabolic burden faced by affected children. Together, these works underscore the importance of addressing environmental and lifestyle drivers to mitigate long-term diabetes risk.

On the genetic and molecular side, a case report by De Souza et al. provided new insights into the clinical manifestations of patients carrying glucokinase (GCK) variants. This work highlighted the heterogeneity of diabetes in children and adolescents, demonstrating how monogenic forms may mimic more common phenotypes of T1D or T2D. Recognizing such genetic causes is essential, as they not only influence prognosis but also guide treatment decisions, sometimes sparing children from unnecessary insulin therapy. This case-based contribution emphasizes the critical importance of genetic testing in refining diagnosis and tailoring clinical care, especially in children with antibody-negative T1D.

On the clinical and management side, Jia et al. investigated mechanistic and integrative insights, reinforcing the central message of this Research Topic: pediatric diabetes cannot be understood through genetics or environment alone. Instead, the disease represents a nexus of inherited risk factors interacting with modifiable exposures such as diet, physical activity, and psychosocial context. This integrative approach is particularly relevant as obesity and other environmental drivers reshape the epidemiology of pediatric diabetes, blurring the lines between T1D, T2D, and monogenic forms.

Taken together, these contributions highlight three major themes. First, modifiable lifestyle factors, including sleep and weight management, remain key targets for prevention and intervention. Second, the identification of genetic variants is essential to resolve the heterogeneous clinical presentations of pediatric diabetes and to enable personalized therapies. Finally, integrating these perspectives points toward a future in which precision medicine can address both the biological and social determinants of health, with the goal of reducing the burden of diabetes in children worldwide.

## Broader context and future directions

The contributions to this Research Topic illustrate how pediatric diabetes sits at the crossroads of genetics, behavior, and environment. Together, they highlight the limitations of a one-dimensional view of disease, instead pointing toward a framework where genetic predisposition interacts with modifiable exposures such as sleep, nutrition, weight status, and psychosocial factors. This multifactorial lens is essential for understanding why diabetes develops in some children but not in others, even among those with shared genetic risk.

Importantly, the articles underscore the urgency of prevention and early intervention. The growing prevalence of T2D in youth, coupled with the persistent burden of T1D, reflects broader shifts in lifestyle and environmental pressures. Obesity, sleep disturbances, and sedentary behaviors act as amplifiers of underlying risk, while genetic heterogeneity adds diagnostic and therapeutic complexity. These findings call for more integrative research approaches that move beyond siloed investigations and instead combine molecular genetics, longitudinal epidemiology, and clinical trial data.

Looking ahead, three priorities emerge. First, identifying reliable biomarkers that bridge genetic and environmental influences could enable earlier risk stratification and preventive strategies. Second, precision medicine approaches must be adapted to pediatrics, where developmental stage and psychosocial context profoundly shape outcomes. Finally, greater attention should be given to the health disparities that amplify diabetes risk in minority and low-income populations, ensuring that new insights translate into equitable advances in care.

By situating genetics and environment within a unified framework, this Research Topic lays the groundwork for the next phase of pediatric diabetes research: one that is preventive, personalized, and equitable.

## Conclusion

This Research Topic emphasizes that pediatric diabetes is neither solely genetic nor purely environmental, but the result of their dynamic interplay. By integrating perspectives on lifestyle, endocrine consequences, genetic heterogeneity, and clinical management, the contributing articles highlight both the complexity and the opportunities for innovation in this field. We thank all authors and reviewers for their contributions, which together pave the way toward more personalized, preventive, and equitable pediatric diabetes care.

